# The pediatric surgeon's perspective on the liver hanging maneuver: a case report and literature review

**DOI:** 10.3389/fped.2025.1536755

**Published:** 2025-03-13

**Authors:** Francesca Gigola, Kejd Bici, Antonino Morabito, Chiara Grimaldi

**Affiliations:** ^1^ School of Pediatric Surgery, University of Florence, Florence, Italy; ^2^ Diagnostic and Therapeutic Services Department, IRCCS-ISMETT (Mediterranean Institute for Transplantation and Advanced Specialized Therapies), Palermo, Italy; ^3^ Department of Pediatric Surgery, Meyer Children’s Hospital IRCCS, Florence, Italy; ^4^ Department of Neuroscience, Psychology, Drug Research and Child Health (NEUROFARBA), University of Florence, Florence, Italy

**Keywords:** liver hanging maneuver, children, hepatoblastoma, hepatectomy, liver resection

## Abstract

**Introduction:**

The liver hanging maneuver (HM) is a well–established technique in hepatic surgery, primarily employed to optimize exposure and simplify parenchymal transection during liver resections. While its efficacy and safety have been extensively documented in adult populations, reports on its application in pediatric surgery are limited. This may be related to peculiarities of the liver anatomy and texture in children and to some specific issues of pediatric liver tumors, especially hepatoblastoma (HB).

**Methods:**

This study reviews the technical adaptations, feasibility, and outcomes of the HM in children, focusing on its role in both routine liver resections and complex cases, such as the separation of conjoined twins. Data of patients treated with and without HM at our center were retrospectively analyzed and a review of recent literature on this topic was performed.

**Results:**

A total of 16 pediatric patients (7 females) underwent HM during hepatic resections with a median age at surgery of 16 months (IQR: 8–22.5). No complications or mortality related to surgery were observed.

**Discussion:**

Results demonstrate that with appropriate modifications, the HM is a safe and effective technique in children, offering advantages in minimizing bleeding while improving surgical efficiency.

## Introduction

Pediatric liver surgery poses distinct technical and anatomical challenges compared to adult cases. Factors such as the small size of pediatric patients, specific issues of pediatric liver tumors like hepatoblastoma (HB), the fragility of the liver parenchyma, and the complexity of vascular anatomy necessitate specialized approaches to ensure safe and effective outcomes. The liver hanging maneuver (HM) technique is widely used in adult liver surgery, mainly for major liver resections like hemi-hepatectomies, but also for other types of segmental liver resections. The technique provides the ability to lift the liver during parenchymal transection by means of a tape passed between the anterior surface of the inferior vena cava (IVC) and the liver.

The HM offers several advantages including better definition of the correct anatomic plan of the parenchymal transection and reduced blood loss. In 2001, Belghiti et al. (1) described this technique to enhance the safety of the anterior approach which was originally proposed by Ozawa to improve postoperative liver function and reduce neoplastic cells seeding (2). In the last decades, modifications of the original technique allowed its adaptation to other types of resections, such as left hepatectomy or posterior sectoriectomy, in both open and minimally invasive approaches.

This manuscript aims to examine the role of the HM in pediatric liver surgery, including the technical modifications required, its application in routine and complex cases, and the associated outcomes. By analyzing available literature and institutional experiences, we seek to demonstrate the feasibility, safety (primary aim), and benefits of this technique in children (secondary aim).

## Materials and methods

We conducted a retrospective analysis of pediatric liver resections performed at our institution from 2016 to 2024 focusing on cases for which HM was utilized. Data were obtained from electronic or physical clinical charts. The Institution's Medical Ethics Review Board recognized that the present study is based on routinely collected information during regular clinical care. No additional data were collected for the analysis. Informed consent was obtained from the patients' legal guardians to publish the intraoperative images. Collected patients' variables included demographic data, primary diagnosis, PREtreatment EXTent of disease (PRETEXT), operative data and complications according to the Clavien-Dindo classification (3, 4).

Furthermore, a comprehensive review of the literature was conducted to identify studies and case reports on the use of the HM in pediatrics. Database search included PubMed, EMBASE and Web of Science, using the following keywords: hepatectomy, hanging maneuver and child or pediatric. We included peer-reviewed English papers reporting hepatic resections using the HM in children (<18 years old). Main reasons for exclusion were adult age, non-English language, and no mention of HM. Data were extracted using a spreadsheet and the following data were analyzed: (1) study characteristics: title, first author, year of publication, number of cases; (2) characteristics of patients and intervention; patients age and weight, tumor location, type of hepatectomy, complications. Data are reported as median and interquartile range (IQR).

## Results

During the study period, seven patients underwent liver resection. Among these, two children were treated using the HM.

### Patients who underwent liver resections without HM

Five patients underwent a liver resection without HM. The median age at surgery was 11 years (IQR: 1.55–15.5), and the indications for surgery included HB (*n* = 1), HCC (*n* = 1), hepatic angiomyolipoma associated with a TSC-2 mutation (*n* = 1), large β-catenin mutated hepatic adenoma (*n* = 1), and hepatic mesenchymal hamartoma (*n* = 1).

Three procedures were performed with an open approach: 2 left hepatectomy, and one bisegmentectomy (segments 5 and 6). In 2 cases, the Da Vinci Xi robotic system was used: one right hepatectomy and one bisegmentectomy (segments 6 and 7).

Median blood loss was 250 ml (IQR: 125–350), and the median length of hospital stay was 8 days (IQR: 7–10). Postoperative complications were observed in two patients: one developed a postoperative fever that was successfully treated with antibiotics and one had a perihepatic hematoma with blood-loss induced anemia successfully treated with packed red blood cell transfusion (Clavien-Dindo2).

### Patients who underwent liver resection with HM

#### Case 1

A 6-years old girl was admitted to the hospital suffering from abdominal pain and was further diagnosed with HB. The abdominal CT-scan and MRI showed a mass in the right lobe of the liver (11.5 × 17.8 × 12.5 cm). A US-guided fine needle core biopsy identified the lesion as HB, alfa-fetoprotein (AFP) was 56.6 UI/ml (normal range: 0.5–5.9 UI/ml). PRETEXT was 3 High-Risk and the patient was treated according to the SIOPEL-4 protocol. After chemotherapy the patient underwent a right hepatectomy. At laparotomy, the liver surface was exposed. A tape was then placed in the space between the IVC and the liver to accomplish the HM. The right hepatic pedicle was identified and clamped. The parenchymal transection was then performed by ultrasound dissection with Cavitron Ultrasonic Surgical Aspirator (CUSA ®, Integra Lifesciences Corporation, NJ, USA). Total clamping time by intermittent Pringle maneuver was 13 min. Surgical time was 328 min; estimated blood loss was 233 ml. No perioperative complications occurred. The postoperative course was uneventful, and the patient was discharged on post-operative day (POD) 6.

#### Case 2

**Figure 1:**
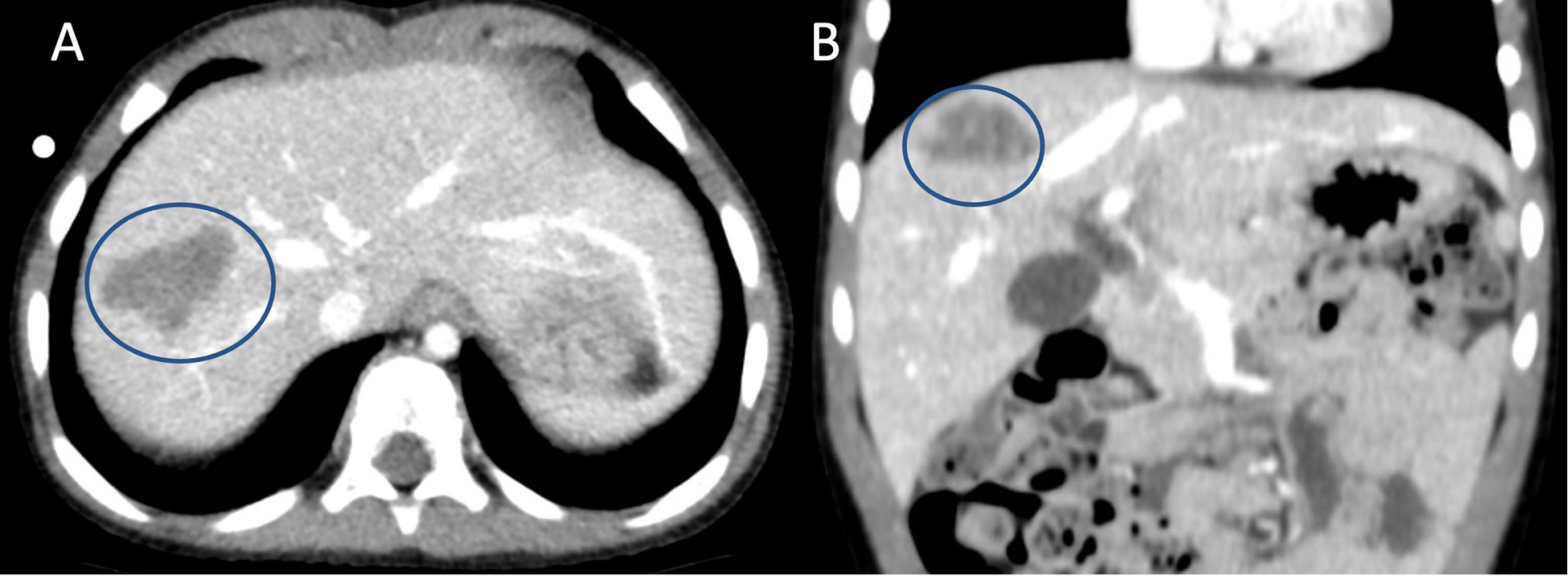
Preoperative CT-scan showing the hepatoblastoma (encircled by the blue line) located in segment 7 near the right hepatic vein. **(A)** axial view, **(B)** coronal view.

A 3-years old boy was diagnosed with HB after being admitted to the emergency department for abdominal pain. AFP was 415 UI/ml (normal range: 0.5–5.9 UI/ml). An abdominal CT-scan showed a mass in the right liver involving segments VII and VII (5.3 × 4.5 × 5.5 cm) and lung metastasis. Percutaneous fine-needle biopsy demonstrated a HB. PRETEXT was 2 HR due to presence of lung metastases and the patient was treated according to the SIOPEL-4 protocol. Preoperative imaging showed an excellent response to chemotherapy with massive tumor shrinkage and clearance of lung metastases (Figure 1).

**Figure 2:**
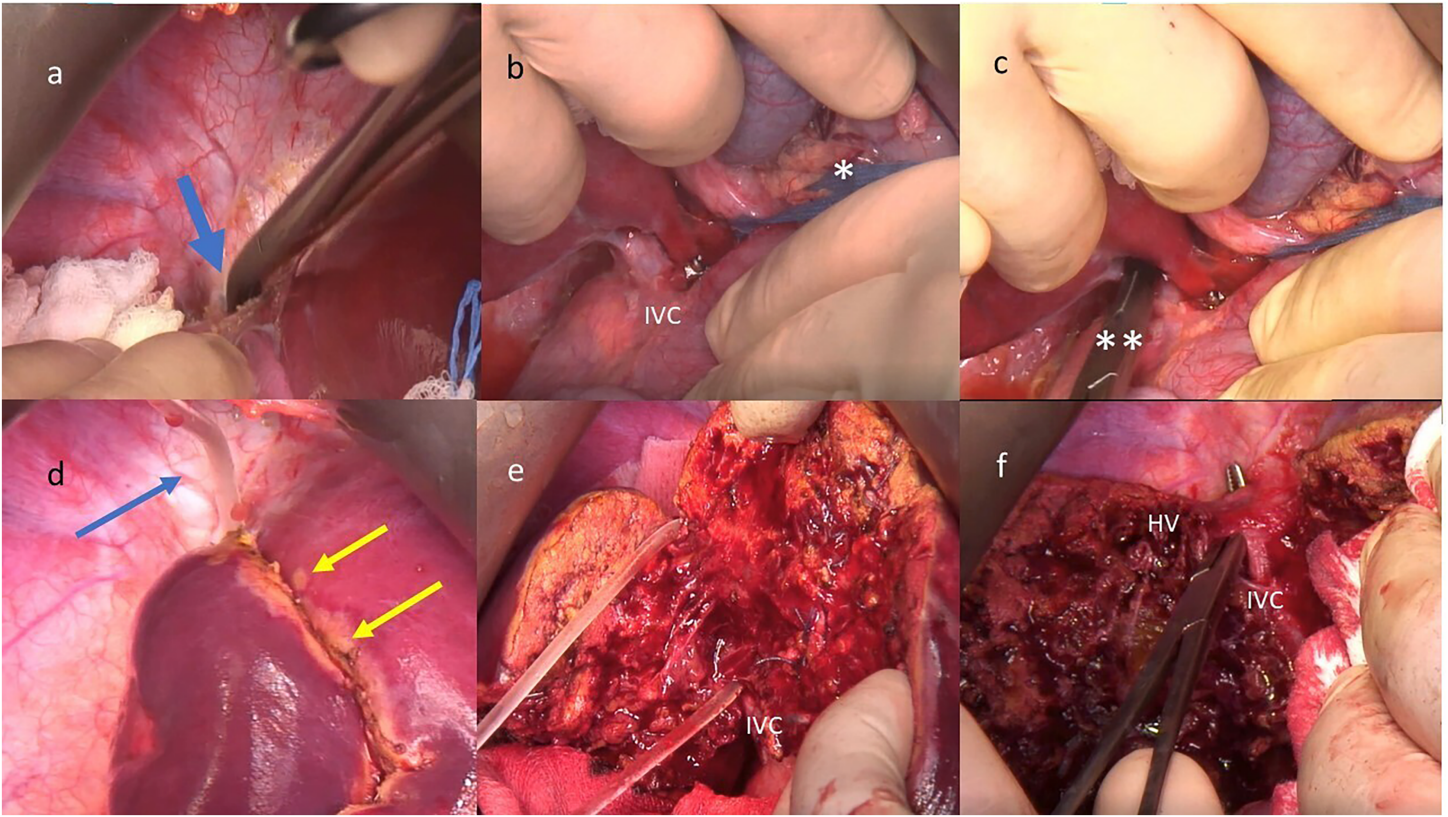
Step-by-step preparation for the hanging maneuver (HM): **(a)** creation of the cranial passage between the retro hepatic inferior vena cava (IVC) and the right hepatic vein (HV), blue arrow; **(b)** preparation for the pringle maneuver (*) and dissection of the anterior aspect of the IVC with specific attention in performing a careful dissection to avoid lesions to the tiny vessels that drain the caudate lobe; **(c)** creation of the caudal passage between the infra hepatic IVC and the liver by a blunt instrument (**); **(d)** upper end of the nasogastric tube used for the HM (blue arrow) and definition of transection line on the liver capsule (double yellow arrow); **(e)** the deep transection plane is lift with the HM, **(f)** At the end of the parenchymal transection the right HV is prepared for ligation.

At surgery, resection of the right posterior sector of the liver was performed after definition of the transection line with the assistance of the HM. A step-by-step description of the main aspects of the surgical procedure is detailed in Figure 2. Operative time was 275 min, estimated blood loss was 130 ml. No perioperative complications occurred; the patient was discharged on POD 6.

### Literature review

The literature search identified 84 studies (23 from Medline, 44 from EMBASE and 17 from Web of Science). Following the removal of duplicates (*n* = 28) and the selection based on title and abstract and full-text, 7 manuscripts met the inclusion criteria (5–11). Two consecutive cases from our database were analyzed and compared with the data of the patients reported in the literature. Patients' data are summarized in Table 1.

**Table 1:** Patients’ characteristics.

First author and year of publication	Cases	Age (m)	Gender	Weight (kg)	Tumor location	Type of hepatectomy	Operative time (min)	Blood loss	Complications
Mochizuki et al., 2011 (5)	3	12	Male	8.4	Right lobe	Right hepatectomy	178	420 g	None
		17	Male	8	Right lobe	Right hepatectomy	330	160 g	
		18	Male	9.2	Left lobe	Left hepatectomy	368	125 g	
Kobayashi et al., 2012 (6)	1	5	Female	7	Right lobe	Right hepatectomy	242	86 ml	None
Ramachandra et al., 2017 (7)	1	8	Male	NA	Right lobe	Right extended hepatectomy + partial resection of segment 1	210	100 ml	None
Nazir 2018 (8)	3	3 days	Male	NA	Segments II and III	Left hepatectomy	NA	NA	None
		4	Female^a^	9.1	Fusion between right and left lobes of the liber with independent biliary tracts	Liver separation of thoraco-omphalopagus conjoined twins	NA	<20 ml	None
		4	Female^a^						
Ramachandra et al., 2019 (9)	1	16	Male	8	Segments V, VIII and IV	Central hepatectomy	320	150 ml	None
Honda et al., 2023 (10)	3	8	Male	6.9	Right lobe + Segment IV	Right extended hepatectomy	476	277 g	None
		21	Male	3.2	Right lobe + Segment IV	Right extended hepatectomy	469	24 g	None
		12	Female	9.2	Segments IVa, VII and VIII	Parenchymal sparing anatomical liver resection	498	57 g	None
Tendean et al., 2023 (11)	2	24	Female^a^	NA	Fusion between segment II and III of “twin A” and II and Iva of “Twin B”	Liver separation of thoraco-omphalopagus conjoined twins	32	NA	None
		24	Female^a^	NA			32	NA	None
Meyer's Children Hospital, 2024	2	36	Male	11	Right lobe	Resection of segments VII & VIII	275	130 ml	None
		72	Female	19.6	Right lobe	Right hepatectomy	328	233 ml	None

m, months; min, minutes; NA, not available.

^a^ Conjoined twins.

Overall, 16 pediatric patients (14 from the literature, 2 from the current series) underwent HM during hepatic resections. Surgery involving HM was performed on 12 patients for oncological reasons, and all of them presented with HB. Additionally, in four patients (two sets of thoraco-omphalopagus conjoined twins), HM was used to facilitate liver separation (patients were 4 months old in one case and 2 years old in the other). Median age at surgery was 16 months (IQR: 8–22.5) and median weight at surgery was 8 kg (IQR: 7.75–8.75). The oldest patient was 6 years old, the youngest was 3 days old and was the only newborn in this series. Median operative time was 320 min (IQR: 210–368) and median estimated blood loss was 127.5 ml (IQR: 78.75–178.25). No complications and no mortality related to surgery were observed.

## Discussion

While granting adequate resection margins, key elements of hepatectomies should be (1) minimizing intraoperative bleeding and (2) preserving the function of the remaining liver (12). To achieve this objectives, Belghiti introduced the HM in 2001, combining it with the anterior approach: this has since been regarded as a significant technical innovation in HPB surgery (1, 2). The original technique involves creating a tunnel between the right anterolateral aspect of the IVC and the liver inferiorly, and then between the right and middle hepatic veins superiorly, to suspend the liver (13). Traditionally, this tunnel is created in a caudo-cranial direction (down-to-up) by blind dissection from both ends of the retro-hepatic IVC (14). While the HM introduces risks, such as injury to the caudate's short hepatic veins during blind dissection of the IVC's anterior surface, careful preparation of the avascular plane can mitigate these concerns and preserve the maneuver's benefits (15, 16). A recent meta-analysis, comparing outcomes of major hepatectomies combining the anterior approach with HM vs. conventional liver resections across 1,109 patients in 16 studies, showed improved perioperative outcomes, including reduced transfusion rates, shorter transection times and hospital stays, and fewer complications (17, 18). Additionally, this combined techniques can enhance postoperative liver function as well as oncological outcomes (13, 14, 19, 20). The major advantages of the HM are evident when parenchymal transection occurs before clamping and sectioning of vascular and biliary structures, such as in living donor liver transplants, resections for hilar cancer, and ALPPS procedures (Associating Liver Partition and Portal vein Ligation for Staged Hepatectomy) (13, 15, 21–25). The HM is widely used by adult HPB surgeons, particularly in right hepatectomies (both open and laparoscopic). However, its use in pediatric resections is rare. Reports on its feasibility and safety in children are limited, though the current literature review shows that expert HPB surgical teams can successfully use the technique in young children with good outcomes. While no reports describe using the maneuver in children younger than five months, it has been successfully reported in a 3.2 kg infant (9). The application of the HM in children necessitates specific modifications to address the smaller anatomical structures and the delicate nature of liver parenchyma. Key adaptations include pediatric vascular clamps or a surgical probe with smaller tapes to safely pass between the IVC and liver parenchyma (5); gentle traction is crucial to avoid parenchymal tearing or vascular injury, in particular to the tiny veins that drain directly into the IVC. Literature reports showed the application of the HM in a child with several comorbidities including trisomy 18 and pulmonary hypertension with the need to prevent elevation of central venous pressure (10).

The primary indication for HM in children is the resection of hepatoblastoma (HB). The rational for introducing this technique is related to the evidence that HBs are typically very large tumors requiring extensive liver mobilization within a limited abdominal space. These anatomical characteristics favor the use of an anterior approach, which can benefit, as described, from the HM. However, the thin parenchymal transection plane and the more superficial position of the inferior vena cava (IVC) in children facilitate better bleeding control, reducing the relative advantage of the maneuver compared to adults (5). Despite the inherent risks of bleeding or vascular injury associated with a blind maneuver in the context of a frail liver parenchyma in small children, no complications were observed in our cases or reported in the literature. This confirms the feasibility and safety of the technique when specific attention is given to technical details, like careful dissection to avoid lesions to the tiny vessels that drain the caudate lobe. When comparing patients who underwent HM to those who underwent liver resection without HM at our center during the study period, we did not observe significant differences in outcomes and complications in the two groups, even though one patient from the “classic” approach group developed a peri-hepatic hematoma necessitating blood transfusion in the immediate post-operative period. HM in pediatric patients may bring additional advantages in the approach to large tumors which distort the intrahepatic vascular and biliary anatomy. Moreover, the HM helps limiting transfusion needs in major hepatectomies by exerting traction and compression on the vessels, thus lowering the risk of major vessel injury (hepatic veins or IVC) (17). Our experience aligns with this finding, as we observed a higher median blood loss in the non-HM group compared to the HM group, however, this difference may also be attributed to a higher median weight at the time of surgery and to different underlying conditions. Furthermore, the limited number of patients included in this study prevents from drawing definitive conclusions.

Since its introduction, several modifications of the original technique have been proposed for different types of liver resections (23). One innovative application of the HM in pediatric surgery is its use during parenchymal transection in the separation of conjoined twins. This complex scenario demands advanced surgical expertise. Reports on the use of the HM in such unconventional cases highlight its potential to address distorted anatomy and limited liver mobilization, optimizing the benefits of the anterior approach. The two reports of the use of the HM in this context, involving two sets of omphalopagus conjoined twins with hepatic fusion, showed how this technique was paramount in providing better anatomical visualization: by suspending the liver during the transection process, the maneuver provided enhanced visualization of the fused hepatic tissue and surrounding vascular structures, minimizing the risk of bleeding and other complications.

Despite its advantages, the HM has some limitations. Pediatric HPB surgery needs great surgical expertise and familiarity with the distinctive characteristics of the liver in children, hence the application of this technique should be limited to expert centers. Furthermore, the availability of specialized instruments may be limited in certain centers, potentially restricting its use. Long term data on the outcomes of HM in pediatric patients are still not available: multicentric studies are necessary to further expand and validate the results of this review, with the need for developing standardized protocols for the application for the HM in pediatric HPB.

## Conclusions

Liver surgery in children should be limited to experienced teams, due to the high technical complexity and the intrinsic fragility of liver parenchyma in small children. The HM is a versatile and effective technique, offering advantages in terms of exposure, bleeding control, and safety. Its application in both routine and complex pediatric cases, such as conjoined twins separations, demonstrates its potential to address some unique challenges of pediatric surgery. With appropriate modifications and expertise, the HM can be safely integrated into pediatric surgical practice.

## Data Availability

The original contributions presented in the study are included in the article/Supplementary Material, further inquiries can be directed to the corresponding author.
